# Supplementary material for: mTOR Inhibition Is Effective against Growth, Survival and Migration, but Not against Microglia Activation in Preclinical Glioma Models
Source: Int J Mol Sci. 2023 Jun 7;24(12):9834. doi: 10.3390/ijms24129834 (PMC10297872; doi:10.3390/ijms24129834)
Supplement: Supplementary file 1 [file ijms-24-09834-s001.zip › ijms-2340202-supplementary.pdf]

FIGURE SUPPLEMENTARY S1

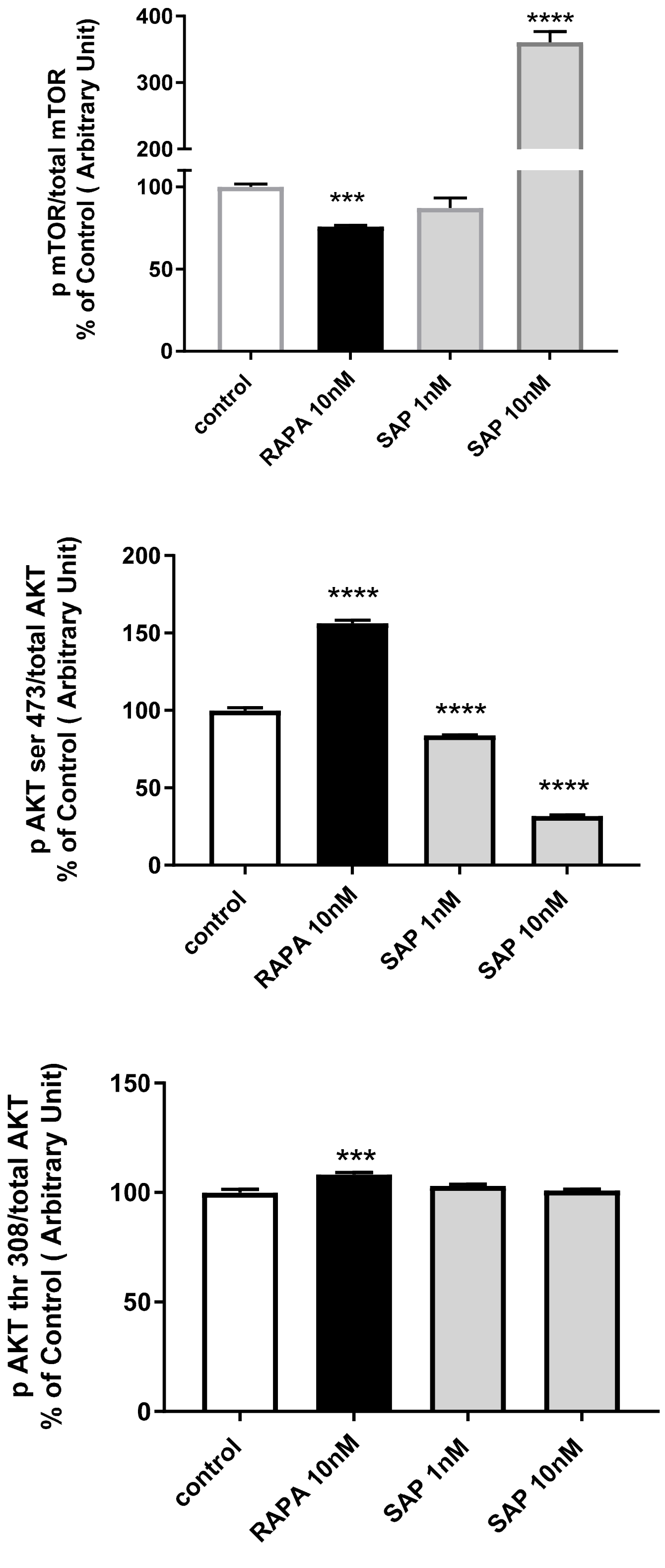

Supplementary Figure S1. Densitometry of pmTOR and pAKT after the following treatments: Lane 1, control, Lane 2, Rapamycin 10 nM, Lane 3, Sapanisertib 1 nM, Lane 4, Sapanisertib 10 nM. Data are means ± SEM, and were analyzed by one-way ANOVA, followed by Dunnett's post-test. *** $p < 0.005$, **** $p < 0.001$.

FIGURE SUPPLEMANTARY S2.

Antiproliferative effects of Rapa and Sap association on U87MG.

A

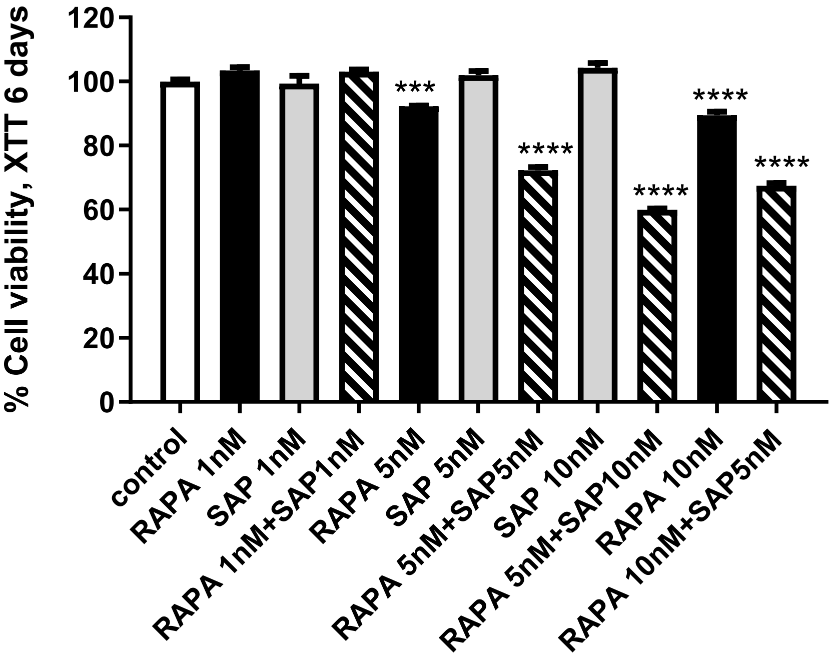

B

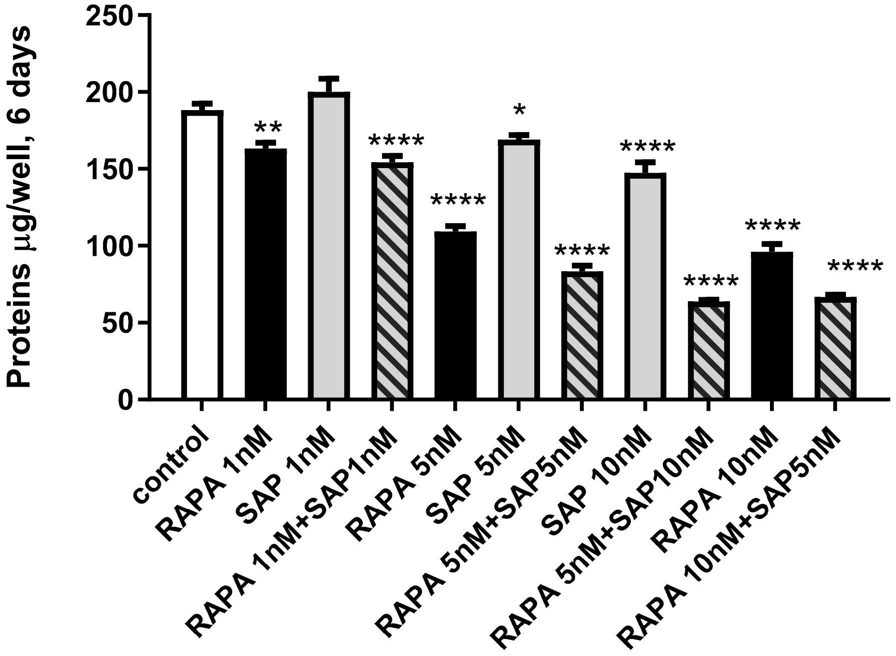

Supplementary Figure S2. Evaluation of treatment toxicity and the effect on cell survival in U87MG. Effect of Rapa, 1-5- or 10 nM, and SAP, 1-5- or 10 nM on cell viability at 6 days by XTT assay (panel **A**). Data are expressed as a percentage relative to the untreated cells (control = 100%) and are means ± SEM. One-way

ANOVA analysis, followed by Dunnett's post-test, was conducted. ***$p < 0.005$, **** $p < 0.0001$. All p values were calculated versus control sample. Panel **B** shows total protein µg per well at 6 days. Data are means ± SEM and one-way ANOVA analysis, followed by Dunnett's post-test, was carried out. * $p< 0.05$, ** $p <0.01$, **** $p < 0.0001$. All p values were calculated versus control sample.

In order to study additive or synergistic effects of mTOR inhibitors, co-administration experiments were conducted for 6 days of treatment. Rapa and Sapa were administrated at doses 1-5 and 10 nM. No significant additive or synergistic effect has been demonstrated.

FIGURE SUPPLEMANTARY S3.

Effects of mTOR inhibitors on T98G cells.

The main experiments carried out on U87MG cells were repeated in another human glioma cell line, T98G. Overall, functional data were confirmed, although the time-course of effects was different. Moreover, Rapa tended to show lower efficacy than SAP in this line. As far as the effects on cell viability are concerned, the overall effects of Rapa and SAP were slower: 100 nM SAP required 6 days of exposure to achieve a significant reduction compared to control, whereas rapa only tended to reduce viability, without reaching a significant effect (Fig 1A). Both Rapa and SAP induced a significant reduction in protein content, but again 6 days of treatment were required (Fig 1B). Looking at LDH release, the results on T98G cells were similar to those obtained with U87MG: 100 nM SAP increased the ratio of extracellular LDH on total LDH, whereas Rapa failed to modify LDH release (Fig 1C).

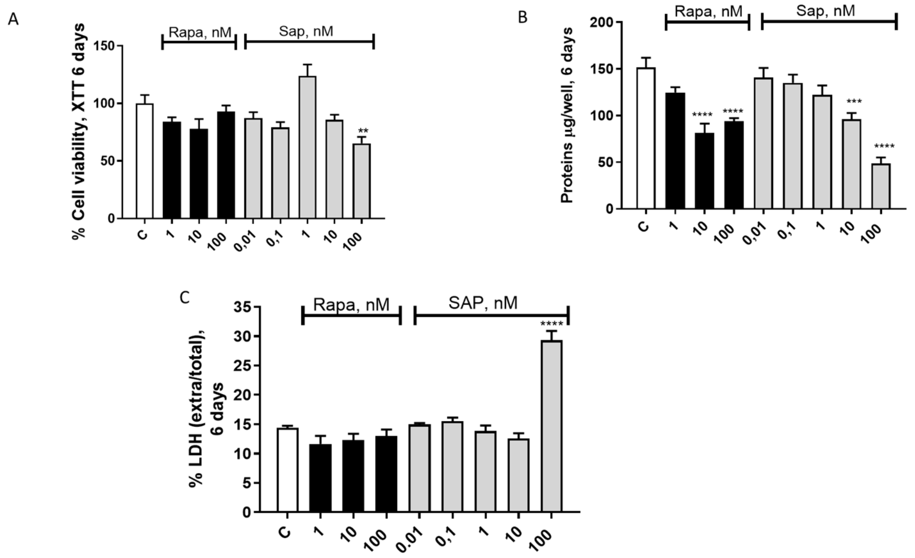

Supplementary Figure S3. Effect of SAP and Rapa on cell survival and its potential toxicity in T98G carried out by measuring extracellular/total LDH ratio (panel **C**), protein and formazan amount (panel **B** and **A**, respectively). All results refer to 6 days of treatment with Rapamycin, ranged from 1nM to 100nM, and Sapanisertib, ranged from 0.01nM to 100nM.Data are means ± SEM and were analyzed by one-way ANOVA, followed by Dunnett's post-test,. ** $p < 0.01$, ***$p < 0.005$, **** $p < 0.0001$.

FIGURE SUPPLEMANTARY S4

Looking at cell migration, the effects of both mTOR inhibitors were similar in the two cell lines. In fact, 10 nM of Rapa and SAP were able to significantly reduce the number of migrating cells (Figure 2).

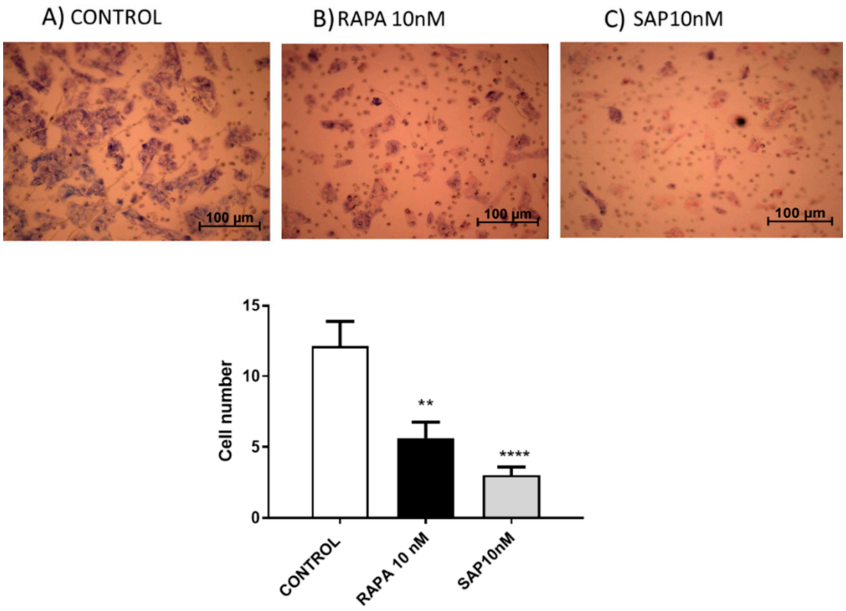

Supplementary Figure S4. Effect of Rapamycin and Sapanisertib on T98 migration and the potential role on reducing tumor invasiveness. Panel **A**, **B** and **C** show the untreated cells, cells treated with 10nM Rapamycin and cells treated with Sapanisertib 10nM, respectively. Cell number count was calculated as mean ± SEM and one-way ANOVA analysis, followed by Dunnett's post-test, was carried out. * $p< 0.05$, ** $p <0.01$**.**

FIGURE SUPPLEMANTARY S5

The two drugs also affected metalloproteinase gene expression, but again 6 days of exposure were required to observe the effects. In particular, Rapa and SAP were able to significantly reduce the gene expression of both ADAM 10 and 17 at all concentrations tested (Figure 3A,B).

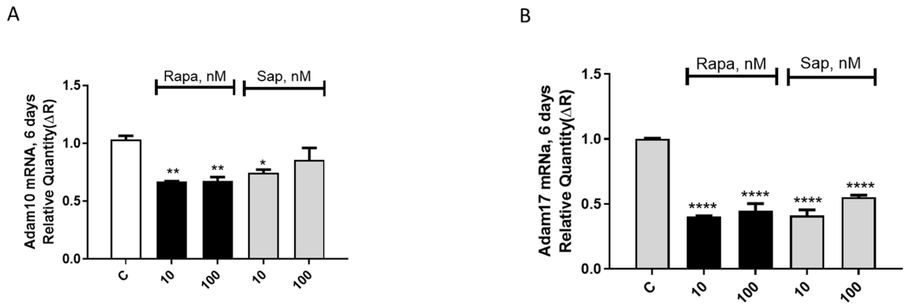

Supplementary Figure S5. Evaluation of Adam 10 (panel **A**), Adam 17 (panel **B**) mRNA level expression in T98G treated with Rapamycin and Sapanisertib, both at 10 and 100 nM. Results refer to 6 days of treatment. Data are expressed as fold change of treated samples versus control, considered as calibrator. Data are means ± SEM, and were analyzed by one-way ANOVA, followed by Dunnett's post-test. * $p< 0.05$, ***$p < 0.005$, **** $p < 0.0001$**.**
